# Rabies Control and Treatment: From Prophylaxis to Strategies with Curative Potential

**DOI:** 10.3390/v8110279

**Published:** 2016-10-28

**Authors:** Shimao Zhu, Caiping Guo

**Affiliations:** Shenzhen Weiguang Biological Products Co., Ltd., Shenzhen 518107, China; gcpzxy0402@aliyun.com

**Keywords:** rabies, rabies vaccine, live-attenuated viruses, monoclonal antibody, nucleic acid-based vaccine, anti-viral therapy

## Abstract

Rabies is an acute, fatal, neurological disease that affects almost all kinds of mammals. Vaccination (using an inactivated rabies vaccine), combined with administration of rabies immune globulin, is the only approved, effective method for post-exposure prophylaxis against rabies in humans. In the search for novel rabies control and treatment strategies, live-attenuated viruses have recently emerged as a practical and promising approach for immunizing and controlling rabies. Unlike the conventional, inactivated rabies vaccine, live-attenuated viruses are genetically modified viruses that are able to replicate in an inoculated recipient without causing adverse effects, while still eliciting robust and effective immune responses against rabies virus infection. A number of viruses with an intrinsic capacity that could be used as putative candidates for live-attenuated rabies vaccine have been intensively evaluated for therapeutic purposes. Additional novel strategies, such as a monoclonal antibody-based approach, nucleic acid-based vaccines, or small interfering RNAs (siRNAs) interfering with virus replication, could further add to the arena of strategies to combat rabies. In this review, we highlight current advances in rabies therapy and discuss the role that they might have in the future of rabies treatment. Given the pronounced and complex impact of rabies on a patient, a combination of these novel modalities has the potential to achieve maximal anti-rabies efficacy, or may even have promising curative effects in the future. However, several hurdles regarding clinical safety considerations and public awareness should be overcome before these approaches can ultimately become clinically relevant therapies.

## 1. Introduction

Rabies is an ancient neurological disease caused mainly by the rabies virus (RABV) and is almost invariably fatal once clinical symptoms develop. Currently, rabies continues to pose a serious public health threat in most areas of the world, especially in the developing countries of Asia and Africa. It has been estimated by the World Health Organization (WHO) that more than 55,000 annual human deaths are caused by rabies, through bites of rabid animals, worldwide. After the virus has entered the periphery site after exposure, it subsequently spreads into the central nervous system (CNS), causing neuronal dysfunction, which is most likely the main cause of the fatal outcome of rabies.

The causative agent RABV is the type species of the genus *Lyssavirus* in the family *Rhabdoviridae*. The RABV genome is a single-stranded, negative-sense RNA of approximately 12 kb, which encodes five structural proteins in the order (3′ to 5′) nucleoprotein (N), phosphoprotein (P), matrix protein (M), glycoprotein (G), and RNA-dependent RNA polymerase (L) [1]. The negative-sense RNA genome is tightly encapsidated by N, P, and L proteins to form a ribonucleoprotein complex that is responsible for virus replication in the cytoplasm within infected cells. The RABV G protein is the only viral protein exposed on the surface of the virus and is not only the major determinant of viral pathogenicity, but also the major protective antigen responsible for inducing protective immunity against rabies [2,3,4,5].

Fortunately, rabies can be a vaccine-preventable disease, provided that post-exposure prophylaxis (PEP) is given promptly and correctly. Protection against rabies correlates with the presence of rabies-specific virus-neutralizing antibodies (VNAs). According to the WHO, VNA titers greater than 0.5 international units per mL serum can reliably provide protection to humans and animals. Currently, rabies-infected dogs are the major reason for the high incidence of human rabies, and therefore vaccinating dogs has been shown to be the most cost-effective strategy for preventing rabies in humans. As reported by the WHO, vaccination coverage of 70% of the canine population can efficiently reduce virus transmission and prevent human rabies. However, despite the fact that efficacious vaccines are readily available, rabies still has a high death rate, mainly due to the cost and accessibility of proper PEP treatment. The current PEP schedule not only requires multiple injections but is also time-consuming, a problem that is even more pronounced due to the fact that RABV-specific immunoglobulin (RIG), which is both expensive and often in short supply, is required to treat severe exposure. This creates a particular burden for rural regions of developing countries that suffer from the highest incidence rates of rabies. Therefore, the development of alternative, cost-effective vaccines that would induce sustained immunity after a single dose inoculation and could ideally clear virus infection from the CNS is warranted.

The advent of the reverse genetics technique has revolutionized the study of RABV, as well as other negative-strand RNA viruses, which has greatly advanced our understanding of the biology of these viruses and profoundly accelerated the development of novel vaccines against various pathogens [6]. While a number of excellent reviews have been written focusing on reverse genetics of negative-strand RNA viruses, the biology of RABV, rabies vaccines, and the pathology and prophylaxis of rabies [6,7,8,9,10], this review rectifies the lack of focus on current strategies that have been evaluated for prevention or as PEP of rabies. This review places particular emphasis on the most promising approaches using live-attenuated and/or recombinant vaccine platforms for preventive vaccinations. Other innovative modalities, such as monoclonal antibody-based platforms and small interfering RNAs (siRNAs) interfering with virus replication, which may deserve future research for rabies treatment, will also be briefly introduced.

## 2. Brief History of Classical Rabies Vaccines

Rabies is one of the oldest infectious diseases and has been known to mankind for more than 4000 years [11]. However, it was not until 1885 that Louis Pasteur developed the first RABV vaccine from the spinal cord of rabbits infected with rabies that was air-dried for inactivation. As the vaccine used by Pasteur was virtually a mixture of inactivated and live RABV, although occasional failures were expected, Pasteur’s vaccine was doomed to be subject to criticism [12]. To solve these safety issues, the Semple rabies vaccine was developed by adding phenol to partially or completely inactivated live viruses in Pasteur’s vaccine [13]. Unfortunately, both Pasteur’s vaccine and the Semple rabies vaccine are derived from nerve tissue and the presence of the myelin component and other potential allergic materials in infected brains restricted the application of these vaccines, due to severe adverse effects. Subsequently, it was found that these issues could be circumvented by producing vaccines from the brain tissue of newborn suckling mice, as the substances responsible for these side effects were largely absent in embryonic and newborn animal nerve tissues. The Fuenzalida rabies vaccine was thereby developed using this technique [14]. However, the vaccine was not fully free of brain tissue components such as myelin, and severe adverse reactions were still reported [15]. An alternative approach to overcome these issues employed embryonated eggs, such as chick or duck embryos, as the media to produce rabies vaccines [12]. Collectively, although these approaches slightly improved the quality of vaccines, they failed to thoroughly resolve the safety issues and were generally less efficacious with poor immunogenicity, which significantly hampered the generalization of these vaccines, and therefore lead to their discontinuation in most areas of the world [12].

The recent advance of modern cell cultivation techniques has made it feasible to produce high-quality rabies vaccines from cell culture. An important development was that, although RABV is highly neurotropic, it can lose its tissue tropism and adapt to in vitro cultured cells, a feature that can be utilized to propagate RABV in many different cell types, in order to achieve high virus yields. The first licensed human rabies vaccine developed from cell culture was the primary hamster kidney cell vaccine, which was created by cultivating viruses in primary hamster kidney cells [16,17]. Subsequently, fixed RABV was adapted to the human diploid cell strain, initially using the lung-derived cell line WI-38, but subsequently switched to the fetal lung cell strain MRC-5, to produce the human diploid cell vaccine [18,19,20]. As the first purified, concentrated, and lyophilized rabies vaccine, the human diploid cell vaccine elicited significantly higher immunogenicity and caused much fewer adverse effects compared to other rabies vaccines, and was therefore recommended as the gold standard reference vaccine by the WHO. However, the lower virus yields and higher production costs make the human diploid cell vaccine difficult to scale up and can be generally unaffordable to most developing countries, where the majority of human deaths from rabies occur. As an alternative, other cell culture vaccines, such as the purified duck/chick embryo cell vaccine, were developed and proved to be as effective as human diploid cell vaccine, and are now commonly used for human rabies prevention worldwide [21,22,23]. Nevertheless, as the primary culture cells inherently have a limited capacity to divide, they are technically difficult to adapt to large-scale industrial cultivation for vaccine manufacturing. For this reason, the Vero cell line was used to produce purified Vero cell rabies vaccine [24]. Vero cell cultivation can easily be scaled up and the virus titers produced in Vero cells are generally higher than those in primary culture cells. Importantly, the Vero cell line has a long history of being used in vaccines without safety concerns [25]. These advantages of Vero cells significantly reduce the costs of rabies vaccine production and make rabies vaccines affordable to most developing countries. Undoubtedly, Vero cells will continue to be one of the most common and popular media for human rabies vaccine production in the future.

## 3. Live-Attenuated Virus-Based Rabies Vaccines

Unlike most other vaccines, however, rabies vaccines are designed to be prophylactic and administered primarily in a post-exposure manner. Due to the extremely high mortality of rabies, only inactivated rabies vaccines were approved for human rabies control. These classic inactivated rabies vaccines are fully protective against rabies, but only when administered pre-exposure or promptly post-exposure and generally given as multiple doses. Once RABV enters the CNS or the symptoms develop, for example in the case of delayed vaccine inoculation, these inactivated vaccines are completely ineffective.

In contrast to inactivated viral vaccines, which can only induce CD4^+^ T cell and humoral responses, live-attenuated viruses could not only induce CD4^+^ T cell and humoral responses but also trigger CD8^+^ T cell responses, as well as innate immune responses that play pivotal roles in cellular immune response against viral infection [26]. In addition, live-attenuated viruses could be produced economically and trigger long-lasting immunity with a single dose, which partially accounts for their wide use as vaccines against many diseases, such as smallpox, poliovirus, and measles [27]. These characteristics of live-attenuated viruses make them ideal candidates for a future rabies vaccine with improved efficacy and potency to fulfill the ultimate goal of a one-dose rabies vaccine for human PEP, and especially benefit infected individuals who would otherwise die from rabies. Currently, a number of recombinant live-attenuated viruses belonging to several families have been investigated as potential vaccines for rabies control (Table 1), and encouraging results were obtained in experimental settings, which could pave the way for future rabies therapy.

### 3.1. Rabies Virus (RABV)-Based Vaccines

Since the first recovery of infectious RABV from cloned complementary DNA(cDNA), using reverse genetic techniques by Schnell et al., numerous studies have been performed to manipulate the RABV genome to combat rabies [40]. An important finding was that the virulent wild-type RABV can be attenuated through serial passages in cultured cells, and subsequent studies have identified the molecular basis for the attenuated phenotype [41,42]. Recent studies have shown that live-attenuated RABV activates, while pathogenic RABV evades, the host innate immune responses in the CNS [43], and it was further demonstrated that co-administration of attenuated RABV with street RABV protects animals against rabies [44]. This can be partially explained by the fact that infection with live-attenuated RABV initially stimulates expression of neuronal cytokines, such as CXCL10, which further attract infiltration of inflammatory cells into CNS and boost induction of proinflammatory chemokines/cytokines, such as MIP-1α and interferon-γ (IFN-γ) in the CNS. This cascade eventually leads to down regulation of the expression of tight junction proteins of the blood–brain barrier (BBB) and thus enhances BBB permeability [45,46]. It has been demonstrated that enhancement of BBB permeability plays a crucial role in the immune clearance of RABV from the CNS, by allowing the infiltration of immune effectors, such as CD4^+^ T cells and VNAs, from the periphery into the CNS to clear viruses, and the failure to open the BBB leads to the lethal outcome of street RABV infection [47]. Interestingly, recent studies have demonstrated that a live replication-deficient RABV-based vaccine can induce both CD4^+^ T cell-dependent and independent B cell activation through the direct infection and stimulation of B cells, which might account for the rapid and potent induction of early protective immune responses by live RABV-based vaccines [48,49]. These studies indicate that it might be possible to develop therapeutics based on live-attenuated RABV for the treatment of clinical rabies. To further increase immunogenicity and address the safety issues associated with a live-attenuated RABV-based vaccine, several strategies have been employed, such as the construction of a replication-deficient virus, targeting specific viral genes, and the insertion of proinflammatory cytokines/chemokines into the viral backbone, using reverse genetic techniques to engineer the RABV genome. These strategies are briefly summarized in Table 2 [9].

In general, live-attenuated recombinant RABV vaccines are not only safe, even in immunocompromised mice, but also elicit high VNA titers and prompt early immune responses against RABV infection [50]. For example, a recombinant RABV expressing the granulocyte–macrophage colony-stimulating factor (GM-CSF) gene payload can prevent mice from developing rabies when administered intracerebrally, at a dose of 10^7.0^ PFU, as late as five days post-inoculation [51].

It should be noted that since most viral pathogens, including RABV, have a complex antigenic structure comprising different proteins that likely all contribute to the induction of a protective immune response, live-attenuated RABVs are probably the most promising candidates for humans as well as animals. Moreover, as the efficacy of vaccination using a specific RABV strain correlates directly with the phylogenetic relationship of the vaccine strain with the circulating virus strains, specific RABV strains that are more closely phylogenetically related with the street isolates circulating in a region might be preferentially selected for rabies vaccine development in a given area. On the other hand, although live-attenuated RABV vaccines are superior in efficacy and are cost-effective for pre- and post-exposure prophylaxis of rabies, compared with conventional inactivated rabies vaccines, live-attenuated RABV vaccines have not been met with wide acceptance. The major concern is safety (i.e., the potential risk of residual virulence and toxicity associated with live-attenuated vaccines, which might cause local reactions, allergic responses, or neurological complications, especially in immune-compromised hosts). Furthermore, the possibility that live vaccines might revert to pathogenic wild-types or even recombine with other live agents, resulting in novel pathogenic agents, cannot be ruled out. Additionally, there is concern about the stability of live-attenuated RABV vaccines. Considering that high temperatures might inactivate or degrade these live-attenuated RABV vaccines, there is a need for the development of appropriate stabilizers that can prevent the inactivation of these live vaccines, such as through the utilization of cold chain during storage and transport. Further studies should focus on analyzing the effect of a combination of these strategies, in order to maximize the anti-rabies efficacy of a live-attenuated RABV.

### 3.2. Vesicular Stomatitis Virus (VSV)-Based Rabies Vaccines

Vesicular stomatitis virus (VSV) is the prototypic species of *Rhabdoviridae* and is one of the best studied animal viruses. Although RABV and VSV are highly similar in their genome composition, the biology of RABV and VSV are quite different. While RABV is a prototype neurotropic virus that causes fatal disease in humans and animals, VSV is an arthropod-borne virus that primarily affects rodents, cattle, pigs, and horses, and most infections of wild-type VSV are non-lethal and only manifest mild symptoms [1]. Importantly, pre-existing immunity to VSV in human populations is generally very low, and VSV infection of humans is essentially asymptomatic and limited to agricultural and laboratory workers [52]. Similar to RABV, numerous strategies, such as gene deletion, have been performed to engineer the VSV genome, in order to increase its immunogenicity and decrease its potential pathogenicity. Along with gene deletion, M gene mutation and gene arrangement greatly advance the use of VSV as viral vectors for numerous problematic viral pathogens.

Interestingly, no studies have been performed investigating the efficacy of using VSV, engineered to express RABV G protein, for protection against rabies. Given the wide application of VSV as a vaccine vector for other pathogenic viruses, we believe that VSV might represent an ideal candidate for rabies control.

### 3.3. Parainfluenza Virus Type 5 (PIV5)-Based Rabies Vaccines

Parainfluenza Virus Type 5 (PIV5), previously known as Simian virus, is a non-segmented negative-strand RNA virus with a genome of about 15 kb in size and belongs to the *Rubulavirus* genus, *Paramyxoviridae* family. It has been shown that PIV5 infects a broad spectrum of cell lines without a significant cytopathic effect, which supports the growth of PIV5 in different cell lines to obtain high viral titers [30]. Importantly, kennel cough vaccines containing live PIV5 have been used in dogs for over 40 years without significant safety concerns for animals or humans, and no association of PIV5 with human diseases has been reported [29]. These features suggest that PIV5 could be used as a good vaccine vector for human rabies control.

It has also been demonstrated that PIV5, expressing the RABV G gene (rPIV5-RV-G), elicits a robust immune response that protects against a lethal RABV challenge [30]. rPIV5-RV-G displayed a dose-dependent protection manner and a single dose of 10^6.0^ PFU of rPIV5-RV-G was sufficient for 100% protection when administered via the intranasal route, while 90% protection was achieved when mice were vaccinated with 10^8.0^ PFU of rPIV5-RV-G via the intramuscular route. Furthermore, mice vaccinated orally with rPIV5-RV-G still showed a significant survival rate, with 50% protection when inoculated with a single dose of 10^8.0^ PFU of rPIV5-RV-G [30]. Most importantly, rPIV5-RV-G protected mice as late as six days after RABV challenge, with 50% protection when inoculated with a single dose of 10^7.0^ PFU of rPIV5-RV-G via the intramuscular route [29]. These results demonstrated that PIV5-G is a promising vaccine candidate for prevention and control of rabies.

### 3.4. Newcastle Disease Virus (NDV)-Based Rabies Vaccines

Newcastle disease virus (NDV) is a member of the genus *Avulavirus* of the family *Paramyxoviridae*, and is one of the virus vectors that are under clinical evaluation for oncolytic cancer therapy, gene therapy, and immune stimulation [53]. One of the obvious advantages of NDV is that it is a bird virus that has adapted only to the avian immune system and does not usually infect mammals due to host range restriction [31]. Several studies have shown that recombinant NDV, expressing the appropriate protective antigens, provided good protection against many pathogen infections, such as influenza virus, human respiratory syncytial virus (HRSV), and severe acute respiratory syndrome (SARS) [54,55,56]. These results indicate that NDV is a promising vaccine vector for humans and other mammals.

A recombinant NDV was constructed, expressing the G gene of the ERA strain (rL-RVG), to evaluate its ability to serve as a vaccine against rabies [31]. It was shown that rL-RVG grow to high titers in cultured cells (up to 10^9.8^ 50% egg infective doses/ml of allantoic fluid) and the expression of RABV G does not change the pathogenicity of the vector virus. Furthermore, intramuscular vaccination with rL-RVG induced a substantial level of VNA production and provided complete protection from challenge with circulating RABV. Most importantly, rL-RVG induced strong and long-lasting protective immune responses against rabies in dogs and cats, with a low vaccine dose of 10^8.3^ 50% egg infective doses completely protecting dogs from challenge with a street RABV for more than a year [31]. These results demonstrated that an NDV-vectored vaccine can be utilized to induce long-lasting systemic protective immunity against rabies in animals and humans in high-risk areas to control rabies infections.

### 3.5. Open Reading Frame Virus (ORFV)-Based Rabies Vaccines

Open reading frame virus (ORFV) belongs to the *Parapoxvirus* genus in the *Poxviridae* family. Due to its very restricted host range to sheep and goats, its skin tropism infection, and the absence of systemic virus spread, even in immunocompromised individuals or after intravenous injection of a high virus dose, a short-term vector specific immunity, and the robust and rapid stimulation of innate immune responses at the site of infection [57,58], ORFV has emerged as a novel virus vector system for expressing different foreign antigens and as vaccine candidates for other pathogenic viruses [32].

It has been shown that a Vero cell culture-adapted apathogenic ORFV strain, D1701-V, expressing the RABV G protein (D1701-V-RabG), correctly presents the RABV G protein on the surface of infected cells without the need for replication or production of infectious recombinant ORFV [32]. Furthermore, one single immunization with D1701-V-RabG stimulated high titers of RABV-specific VNA in mice, cats, and dogs. A single intramuscular inoculation of 10^7.0^ PFU of D1701-V-RabG completely protected mice against an intracranial challenge with high lethal doses of the virulent RABV strain CVS-11 [32]. These results demonstrated that ORFV is a valuable virus vector to be used as vaccines for rabies control and treatment in the future.

### 3.6. Vaccinia Virus-Based Rabies Vaccines

Vaccinia virus is a large, complex, enveloped double-stranded DNA virus belonging to the *Orthopoxvirus* genus in the *Poxviridae* family. Similar to other poxviruses, vaccinia virus replicates uniquely and exclusively in the cytoplasm of the infected cells [59]. Vaccinia virus infection is generally very mild and is typically asymptomatic in healthy individuals. The most famous application of vaccinia virus in modern vaccinology is the successful use of vaccinia virus to eradicate smallpox, which is a milestone in the fight against infectious diseases during the history of mankind, and vaccinia virus is still being used as a live-virus vaccine against smallpox today [60].

Several recombinant poxviruses have been licensed for use as vaccines against numerous pathogen infections, including rabies. The first recombinant poxvirus licensed and used as a vaccine is the recombinant vaccinia virus Copenhagen strain, expressing a RABV G gene, which was inserted into the poxvirus thymidine kinase gene (V-RG) [60]. It has been suggested that oral rabies vaccination may be the only effective strategy for rabies elimination programs in domestic and wildlife animals. As a live virus vaccine, V-RG was one of the two vaccines (the other was the RABV SAG-2 strain) successfully used as oral baits to control rabies in wild animals, such as red foxes, raccoons, and coyotes, in North America and Western Europe [34,35]. However, V-RG failed to induce adequate protection in skunks and dogs when administrated orally as a single dose [61,62]. Moreover, V-RG has been associated with severe skin inflammation in humans who have occasional contact with the baits [63]. Another drawback of V-RG is the pre-existing, or vaccination-induced, vector immunity, which could inhibit the generation of sufficient anti-rabies immune response [64]. As an alternative, a safer, highly attenuated, and host-restricted variant of vaccinia virus, the modified vaccinia virus Ankara (MVA) strain, was developed through serial passages in primary chick embryo fibroblast cell culture [65]. Unfortunately, a recombinant MVA expressing the RABV G gene was shown to be less immunogenic than V-RG, with up to two log titers of the recombinant MVA being required to elicit an equivalent VNA response in laboratory mice [60]. However, the recombinant MVA did elicit adequate protective immune response in dogs and raccoons by oral administration [66]. Other poxviral vectors, such as canarypox virus, raccoon poxvirus, capripoxvirus, and fowlpox virus, have also been used to generate recombinant live vaccines against rabies [60]. These vaccines generally have their limitations and are thus not ideal for use as rabies vaccines.

### 3.7. Human Adenovirus (HAdV)-Based Rabies Vaccines

Adenoviruses are a family of non-enveloped double stranded DNA viruses with an icosahedral nucleocapsid that are pathogenic to a broad range of vertebrate hosts, such as reptiles, birds, mammals, and amphibians [67]. Adenovirus vectors have been shown to be highly efficacious and are capable of inducing strong antibody CD4^+^ and CD8^+^ T cell responses [68], making them ideal candidates for gene therapy and vaccine development. As human adenoviruses are by far the best characterized, most applications of adenoviruses, such as gene transfer studies and vaccine vectors, have been carried out with vectors based on the replication-defective human serotype 5 (HAdV-5), with the essential E1 or E3 gene deleted [69].

An E1-deleted HAdV-5 expressing the RABV G protein, Adrab.gp, was constructed and tested in mice [36,37]. Mice immunized at birth with Adrab.gp developed antibodies to RABV and were protected against subsequent lethal challenges. Furthermore, immune responses elicited by Adrab.gp were not impaired by maternal immunity, suggesting that Adrab.gp could be useful for immunization shortly after birth [37]. It was shown that Adrab.gp induced high titers of VNA against rabies in dogs previously immunized with the conventional rabies vaccine [70]. AdRG1.3, also known as ONRAB^®^, is a replication-competent HAdV-5 vector, with an insertion of the RABV G gene into the E3 region, thus rendering the virus replication defective [71]. It was demonstrated that AdRG1.3 induced high levels of VNAs for protection against rabies, and importantly, AdRG1.3 was immunogenic and demonstrated significant protection as baits both to skunks and raccoons, for which the V-RG vaccine showed limited efficacy [72]. Besides HAdV-5, a number of other adenoviruses, such as the avirulent canine adenovirus type-2 [73], chimpanzee adenovirus vector serotype SAd-V24 (also termed AdC68) [74], and avian adenovirus Celo virus [75], have also been investigated for use as rabies vaccines, and these recombinant adenoviruses generally elicit robust VNA production and provide adequate protection against rabies. These results demonstrated that recombinant adenovirus-based vectors warrant further consideration for human rabies control and particularly for the oral vaccination of wildlife animals.

### 3.8. Pseudorabies Virus (PRV)-Based Rabies Vaccines

Pseudorabies Virus (PRV), also known as Suid herpesvirus 1 or Aujeszky′s disease virus, is a member of herpesvirus type 1 virus, belonging to the *Varicellovirus* genus in the *Alphaherpesvirinae* subfamily of the *Herpesviridae* family. The genome of PRV is a linear, double-stranded DNA enclosed within a protective icosahedral nucleocapsid [76]. PRV is the main cause of Aujeszky′s disease in swine and is endemic in most parts of the world [77]. Recently, PRV has attracted much attention on disease control in swine agriculture, and has also been a useful model for the study of herpesvirus biology and neuronal pathways. In addition, vectors based on PRV have been developed for delivery of viral antigens, to improve the efficacy of vaccines for pig pathogens [78].

A recombinant PRV, expressing the RABV G gene (rPRV/eGFP/rgp), was constructed and analyzed for its efficacy for protection against rabies [38]. It was shown that the growth rate of rPRV/eGFP/rgp was comparable to that of the parent PRV strain. rPRV/eGFP/rgp was safe in dogs by oral and intramuscular routes of inoculation and induced protective immune responses against both pseudorabies and rabies in dogs, via a single oral inoculation of 2×10^7.0^ PFU, with the VNAs produced by rPRV/eGFP/rgp maintaining for over six months [38]. These results demonstrated that PRV may serve as an alternative live vector for the development of an oral rabies vaccine for dogs.

### 3.9. Autographa Californica Multiple Nucleopolyhedrovirus (AcMNPV)-Based Rabies Vaccines

Autographa californica multiple nucleopolyhedrovirus (AcMNPV) is the type species of the *Baculoviridae*, a family of enveloped double-stranded DNA viruses that are specifically pathogenic to arthropods, and mainly affects insects of the orders Lepidoptera, Hymenoptera, and Diptera [79,80]. Due to the strict host specificity, baculoviruses are probably the most beneficial viruses to humans and have been used as bio-insecticides against forestry and agriculture pests and as vectors for virus-like particle expression and surface display techniques [81,82]. Most importantly, the baculovirus–insect expression system is one of the most popular and robust eukaryotic expression systems for heterologous gene expression in insect cells [83].

A recombinant AcMNPV, expressing the RABV G gene, was constructed under the control of the AcMNPV *polyhedrin* and the immediate early CMV promoters [39]. An *in vitro* study demonstrated that BV-RVG/RVG induced syncytium formation in insect cells and displayed efficient gene delivery into mammalian cells. Importantly, mice intramuscularly immunized with BV-RVG/RVG developed high levels of VNAs and were completely protected against lethal RABV challenge [39]. These results demonstrated that baculovirus can be used as an alternative strategy to develop a safe and efficacious vaccine against rabies.

## 4. Other Novel Modalities for Rabies Control and Prevention

Although live-attenuated virus-based vaccines represent the most promising approach for rabies control and treatment, some other novel modalities have also been investigated for their potential role in rabies treatment, such as protein and peptide vaccine, nucleic acid-based vaccine, RNA interference (RNAi), and RIG, coupled with BBB permeability enhancing agents such as monocyte chemotactic protein-1 (MCP-1). Still another promising approach is a bi-specific antibody (BsAb) with one arm targeting a receptor involved in the receptor-mediated transcytosis pathway of the brain while another one targets the RABV G protein, thus potentially enabling efficient transport of antibody across the BBB, in order to clear RABV from the CNS.

### 4.1. Protein Subunit and Peptide Vaccine

Several studies have investigated the efficacy of G protein (purified from infected cells) for rabies protection, based on the fact that RABV G protein is the major determinant of viral pathogenicity and is also the major protective antigen responsible for inducing protective immunity against rabies. In one study, the RABV G protein was engineered into the yeast system and the yeast extracts containing the rabies G protein protected guinea pigs from a lethal RABV challenge when administered intramuscularly, but failed to protect mice via a lethal intracerebral challenge [84]. In another study, Sakamoto et al. showed that most rabies G protein expressed in yeast were not processed correctly and were therefore poorly immunogenic [85], which might be ascribed to the structural complexity and multimeric propensity of the G protein. As mentioned above, a baculovirus-expressed G protein in insect cells was found to be antigenically conserved and immunogenic in mice [86], and provided protection to raccoons against a lethal challenge with a street RABV via repeated oral immunization with G protein-containing cell lysate [87]. Furthermore, there are researchers using plants, such as maize and tobacco, to express RABV G protein to obtain the so-called edible vaccine [88,89,90]. Although these forms of vaccines induced a detectable VNA response and protection against challenge in mice upon ingestion, thus potentially being generated at a low cost, several limitations were obvious, including immunogenicity, the stability of the vaccine in fruit, the degradation of the vaccine in the stomach and the intestinal immune response. Therefore, despite that fact that edible vaccines are attractive and appealing options, much work must be conducted before they can become a viable option for an alternative rabies vaccine. Overall, protein subunit vaccines are often poorly immunogenic and can induce limited immune responses. It should also be noticed that, for therapeutic use of a vaccine given by injection, the expressed proteins must be extensively purified, which would likely render this approach cost-ineffective.

Besides a protein subunit vaccine, peptide vaccines have also been conducted to test their efficacy for rabies protection. A number of antigenic epitopes have been identified in RABV G protein, and peptides mimicking these epitopes have been synthesized. It was shown that a peptide expressing a linear epitope of the G protein (named G5), located at aa 244–281 of the G protein, induces a strong humoral immune response in the absence of a carrier protein in immunized goats, which mounted high antibody titers and showed a strong avidity to both the G5 peptide and the purified surface G protein [91]. However, the antibody produced weakly neutralized RABV carrying the G5 epitope and failed to neutralize escape mutants carrying a single point mutation in this epitope [91]. Peptide mimotopes of the G protein (identified from a random constrained hexapeptide phage display library, using neutralizing human anti-RABV IgG antibodies), were shown to be matched to antigenic site III of the G protein, and these mimotopes were able to interact with the human anti-RABV IgG antibodies in a dose-dependent manner [92]. While subcutaneous administration of the RABV G site III mimotope induced an anti-RABV G-specific IgG response in mice [92], it does not address whether these mimotopes could elicit immune protection against a lethal challenge in a mouse model. On the other hand, a chimeric peptide containing antigenic determinants of the G protein (amino acids 253–275) and N protein (amino acids 404–418) was constructed to fuse with the coat protein of alfalfa mosaic virus in tobacco or spinach plants [90]. The recombinant virus induced an immune response in mice and protected mice against challenge infection: following a single dose of conventional rabies virus vaccine, three of nine individuals showed detectable levels of RABV-neutralizing antibodies, indicating the potential of the plant virus-based expression systems as a supplementary oral booster for rabies vaccinations [90]. However, the high variability of the G protein would compromise these peptide vaccines that induce antibodies to specific epitopes. Still, there are other novel approaches using peptides to target one or multiple critical steps in RABV replication; a case in point are peptides that mimic the RABV P protein, which are a core component of RABV transcription/replication complex [93]. Peptides containing the first 57 or 60 amino acids of P protein were shown to inhibit RABV but not VSV infection of BHK-21-T7 cells [93]. Importantly, these peptides also significantly inhibit virus replication in neuronal cells [93]. These results implied that peptides that interfere with certain steps in viral replication hold promise as a therapeutic approach against rabies.

### 4.2. Nucleic Acid-Based Rabies Vaccines

As the next-generation vaccines, nucleic acid-based vaccines have drawn much attention in the past two decades. Generally, nucleic acid vaccines are based on DNA or RNA that encodes a specific antigen of interest. Nucleic acid-based vaccines possess the capability to mimic a live infection by expressing antigens in situ after immunization without posing safety concerns and induce both humoral and cellular immune responses, and therefore have been widely explored for numerous types of disease control and cancer treatment, especially in circumstances where no vaccine exists or for replacing existing vaccines [94,95,96,97]. Other advantages of nucleic acid-based vaccines include stability, low production costs, ease of development and purification, and scaling up of production being much simpler compared to that of purified protein vaccines or viral vaccines [69].

A number of studies have been conducted to evaluate the efficacy of DNA vaccines to rabies. A plasmid pSG5rab.gp that expresses the RABV G protein, under the control of the SV40 early promoter vector, was tested in mice for induction of rabies-specific immune responses [98]. Mice intramuscularly immunized with the pSG5rab.gp vector developed RABV G-specific cytolytic T cells, lymphokine-secreting T helper cells of the Th1 subset, and rabies VNAs, and were fully protected against a subsequent lethal RABV challenge [98]. A pClneo plasmid encoding the RABV G protein under the control of the CMV promoter (pGPV) was constructed and tested in dogs [99]. Beagles intramuscularly immunized with pGPV developed high VNA titers against wild-type RABV and to a lesser extent, to European Bat Lyssaviruses (EBL1 and EBL2), and all vaccinated dogs were protected against a lethal RABV challenge with a wild-type dog rabies strain [99]. Importantly, a comparison between the DNA vaccine and a traditional human diploid cell vaccine in monkeys showed that these two approaches elicited comparable primary and anamnestic VNA responses, and all vaccinated monkeys survived a lethal RABV challenge [100]. Still, some researchers have explored the use of replicon-based self-replicating DNA vaccines derived from an alphavirus (Sindbis virus), to improve the biosafety and immunogenicity of DNA vaccine [101]. This recombinant replicon expressed rabies G protein efficiently and induced apoptosis in transfected cells, an important event in the enhancement of immune responses [101]. Mice intramuscularly injected with this replicon-based rabies DNA vaccine induced comparable humoral and cellular immune responses, compared to commercial cell culture vaccines, but better than conventional rabies DNA vaccines [101]. In addition, both replicon-based rabies DNA vaccines and commercial cell culture vaccines conferred complete protection against a lethal RABV challenge [101]. These results clearly demonstrated that DNA vaccines are promising candidates for rabies control.

Despite the tremendous advantages of DNA vaccines in mass vaccinations against rabies, several drawbacks of using DNA vaccines need further investigation. The slow onset and modest induction of protective immune responses upon DNA vaccination precludes their use for post-exposure immunization against rabies. This is probably due to the poor immunogenicity and inadequate uptake of DNA vaccines by cells at the inoculation site, thus multiple immunizations of high DNA doses are often required to produce enough antigen for optimal immune responses. A number of improvements have been made to increase the safety and efficacy of DNA vaccines. To improve DNA uptake, electroporation has been used and was shown to generate comparable or superior levels of immune responses [102,103]. To increase the immunogenicity and efficacy of DNA vaccines, several approaches have been conducted, including using chemical adjuvants [104,105], co-administrating plasmids expressing cytokines [106,107], modifying the targeted G protein [108,109], increasing the expression level of encoded proteins [94], co-inoculating with a traditional inactivated rabies vaccine [110], and optimizing DNA vaccine formulation, such as through targeted trafficking of a G protein to a lysosome supplemented with adjuvant Emulsigen-D [111,112]. These endeavors have significantly increased the immunogenicity and efficacy of DNA vaccines and extended their potential in preventing and controlling rabies. However, risks still exist as to the integration of plasmid DNA into a host chromosome, the tolerance to the plasmid DNA vector and the antigen, autoimmunity, and the generation of a human anti-DNA immune response [94,113,114,115]. For example, the integration of DNA into the host genome could result in the potential risk of long-term or even uncontrolled expression of the transgene. Furthermore, the integration of DNA into the host genome may also modify the genetic information of the host and thus be tumorigenic. Therefore, much effort is needed to unravel the action and mechanisms of DNA vaccines before they become a broadly useful vaccine and immunotherapy platform.

Besides DNA vaccines, several studies have investigated the potential of vaccines based on RNA molecules for rabies treatment. Currently, two major forms of RNA vaccines have been developed based on the auto-replicative capacity of messenger RNA (mRNA): conventional non-amplifying mRNA vaccines and self-amplifying mRNA vaccines (also termed replicons) derived from an RNA virus vector such as the Semliki Forest virus (SFV) that maintains replicative activity [116]. As for the conventional non-amplifying mRNA-based rabies vaccines, it has been shown that an mRNA encoding RABV G gene induced potent VNAs, and antigen-specific CD4^+^ and CD8^+^ T cells in inoculated mice [117]. Importantly, a mRNA-based rabies vaccine protected mice against lethal intracerebral challenge infection with an efficacy comparable to a commercial inactivated rabies vaccine. Moreover, the mRNA-based rabies vaccine was also immunogenic in domestic pigs and elicited VNA levels comparable to those induced by full doses of commercial inactivated rabies vaccine [117]. This study demonstrated the promise of non-replicating mRNA rabies vaccines for the prevention of rabies. In the case of self-amplifying mRNA-based rabies vaccines, it was shown that a recombinant SFV, carrying an RNA coding RABV G protein (SFV-RVGP), induced high expression levels of functionally trimeric RABV G protein [118]. Moreover, SFV-RVGP elicited comparable levels of antibodies to that of a commercial rabies vaccine, and was more effective than the protein vaccines in inducing a cellular immune response [119].

While initial studies have used naked mRNA as the vaccine, much effort has been put into enhancing the delivery efficiency and potency of an RNA vaccine. To alleviate the degradation of RNA and enhance cellular uptake, methods such as gene gun, electroporation, liposomes, and cationic polymers have been used to obtain improved delivery systems for RNA vaccines [116,120]. Similar to DNA vaccines, chemical adjuvants, or co-administrating RNA expressing specific adjuvants, have been investigated to enhance immunogenicity [97]. Finally, as to the optimization of mRNA for vaccination, several approaches have been conducted to modify the RNA molecule directly, such as the modification of the 5′ cap structure or the untranslated and the translated regions, to achieve high and long-term antigen expression [121].

Vaccination with RNA vaccines has become an increasingly promising strategy for disease control over the past decade. Compared to DNA vaccines, RNA vaccines are superior in several aspects. Unlike DNA vaccines, which have a potential risk of integration with the host genome, vaccines based on RNA would eliminate this concern. In addition, DNA vaccines must be delivered into and transcribed within the nucleus to express the antigen, which is not only time-consuming but may be inefficient in some types of cells, such as non-dividing mature myocytes. On the contrary, RNA vaccines are directly translated in the cytoplasm, which not only obviates the need for delivery into the nucleus but also leads to rapid antigen expression. Finally, it has long been known that RNA is a potent stimulator of innate immunity through signaling via pattern recognition receptors, such as the Toll-like receptors or the retinoic acid-inducible gene I-like receptors, and this adjuvant effect of RNA stimulates the host immune response [97]. Although it is argued that RNA is generally less stable than DNA, this in turn guarantees the tight control of the immunogenicity of RNA vaccines to efficiently limit vector-derived immunity and other safety issues due to the persistence of RNA. These attributes of RNA vaccines make them an ideally adaptable and versatile vaccine platform for infectious disease control.

### 4.3. Small Interfering RNA (siRNA)-Based Therapy

siRNA, also known as short interfering RNA or silencing RNA, is a class of double-stranded RNA molecules 21–23 bp in length, that functions within the RNAi pathway to interfere with the expression of specific genes with complementary nucleotide sequences by degrading mRNA after transcription, thus leading to the disruption of protein translation and post-transcriptional gene silence [122,123]. Due to its easy manipulation, powerful efficacy, and induction of little cytopathy, siRNA-based gene silencing of target genes has been considered one of the most promising approaches to achieve antiviral defense against various diseases and disorders [123].

Initially, plasmids encoding siRNA, or another form of RNAi that target specific RABV genes, were constructed and tested for anti-rabies efficacy [124,125,126]. Although the plasmid-based approaches achieved some degree of anti-rabies effect, the low efficiency of siRNA delivery hindered the substantial protection against RV through anti-rabies RNAi approach. To increase delivery efficiency, viral vectors, such as adenoviruses [127,128,129] or lentiviruses [130], were used to express these siRNAs. Generally, these viral vector-based siRNAs efficiently inhibited corresponding gene expression and the RABV multiplication and conferred significant protection against lethal RABV challenge. As for silencing efficiency, two RABV genes (the highly conserved N and L genes) were generally chosen for targeting, although the P and G genes were also reported to have antiviral potential. Still, to overcome the safety issues related to viral vectors, a liposome was used to deliver siRNA-encoding plasmid and significantly increased the survival of RABV-infected mice compared to a non-liposome-mediated delivery method [131].

Another promising delivery method has been proposed by Kumar et al., who have demonstrated that a short 29-amino-acid peptide, derived from the RABV G protein, which binds to the acetylcholine receptor exclusively expressed by neuronal cells, enables the transvascular delivery of siRNA across the BBB to the brain [132]. A chimeric peptide, containing nonamer arginine residues added to the carboxy terminus of the 29-amino-acid peptide, was shown to bind and deliver siRNA to neuronal cells which lead to specific gene silencing within the brain in mice after intravenous administration [132]. Furthermore, intravenous treatment with a chimeric peptide-bound antiviral siRNA complex provided robust protection against a fatal Japanese encephalitis virus challenge in mice, and repeated administration of the chimeric peptide–siRNA complex did not induce inflammatory cytokines or anti-peptide antibodies, indicating that this chimeric peptide provides a safe and noninvasive approach for the delivery of siRNA across the BBB [132]. Taken together, these results demonstrated that siRNAs-based approaches have the potential to be developed into new and effective anti-rabies therapy.

### 4.4. RABV-Specific Immunoglobulin (RIG) Coupled with BBB Permeability-Enhancing Agents

RIG, a recombinant monoclonal antibody targeting a specific epitope of the G protein, is highly effective in neutralizing RABV in vitro or before the virus enters the CNS [7]. It is an essential component of PEP, since it delivers passive immunity which is particularly important at early stages, before the host develops active immunity to the vaccine. However, once the virus accesses the CNS, these antibodies are restricted to crossing the immune-privileged BBB to neutralize virus infection. Although, in theory, the antibody could be given intracerebrally, it is definitely not a practical therapy. On the other hand, it has been firmly established that enhancement of BBB permeability is required to allow the passage of VNAs into the CNS of activated B cells, in order to cross the BBB and release antibodies in situ for RABV clearance [133,134,135]. As mentioned above, a live-attenuated virus could transiently enhance the BBB permeability, although this raises safety concerns. An alternative way is to use agents, such as the cytokine MCP-1 or hyperosmotic solution, to enhance the BBB permeability, thus allowing antibodies to pass through the BBB and enter the CNS. It was shown in mice that intravenously administered VNAs are crucial in the clearance of RABV from the CNS and could prevent the development of rabies in both immunocompetent and immunocompromised mice, as long as the BBB permeability is enhanced using MCP-1 [133]. In rats, the breakdown of BBB permeability using hypertonic arabinose, combined with passively administered virus-neutralizing mAb 8-10E, prolonged the survival of infected rats [134]. Interestingly, mAb 8-10E treatment alone partially increased the survival rate of infected rats [134]. These results demonstrate that simultaneous administration of VNAs and BBB permeability-enhancing agents might be a therapy for clinical rabies. However, considering that BBB permeability-enhancing agents might negatively affect the function of CNS integrity through uncontrolled BBB enhancement, precautions should be taken to preserve homeostasis and avoid adding to new neuronal damage of the CNS when using these BBB permeability-enhancing agents.

### 4.5. Bi-Specific Antibody (BsAb)-Based Therapy

BsAb is composed of two binding specificities for two different antigens, or two different epitopes of an antigen, in one antibody molecule. BsAb is a newly emerging area in modern biology and is one of the most promising and exciting advancements in cancer immunotherapy [136]. The purpose of BsAb is to recognize and target two different antigens simultaneously, thus realizing the desired therapeutic effects that cannot be achieved by a combination of two monospecific antibodies [137]. In cancer immunotherapy, one of the most obvious advantages of BsAb is its ability to redirect immune effector cells to the proximity of targeted tumor cells, thus improving the tumor-killing potential of these immune effector cells. This prototypic form of BsAb is generally composed of two linked single-chain fragment variables with one targeting molecule present on immune effector cells, such as the CD3 found on T cells, while the other targets a tumor-specific antigen.

Currently, no studies have been reported that use BsAb to treat rabies. As mentioned above, sufficient levels of VNAs and the enhancement of BBB permeability are critical for the clearance of RABV from the CNS. As live-attenuated viruses pose safety concerns while BBB permeability-enhancing agents can cause unwanted neurological complications due to the disruption of normal BBB integrity, the ideal approach is to deliver VNAs without altering the normal function of the BBB. Endogenous BBB receptors involved in the normal receptor-mediated transcytosis (RMT) pathways that function to transport macromolecules such as insulin and transferrin into the brain are ideal targets that can be utilized to deliver biologics; a case in point are rabies VNAs, which can be delivered to the CNS without interfering with the normal functionality of the BBB. Such receptors include two well-characterized transferrin receptors [138] and an insulin receptor [139], or the recently identified solute carrier CD98 heavy chain (CD98hc) [140]. Therefore, targeting these receptors can act as a molecular Trojan horse to ferry biologics fused to the antibody into the brain via RMT. Consequently, a BsAb for rabies treatment could be constructed with one arm targeting one of the endogenous BBB receptors while the other targets the RABV G protein. Theoretically, this BsAb could pass through the BBB while retaining the ability to neutralize RABV in the CNS, thus potentially indicating a novel therapy for rabies treatment in the future.

## 5. Conclusions

Rabies continues to pose a severe burden to public health and is ranked one of the most fatal diseases. It causes tens of thousands of human deaths annually, particularly in developing countries. Currently, dogs remain the main source of rabies and are responsible for almost 99% of fatal rabies cases in humans. Since the first development of the rabies vaccine by Pasteur, human rabies vaccines have been improved and refined. Current cell culture rabies vaccines for humans are highly efficacious, safe, and easily accessible in most parts of the world, which in turn has enabled the control of rabies in these regions. Therefore, in terms of rabies exposure, as long as prompt and correct PEP are administered, there is no need for further development of rabies vaccine for current PEP. In high-risk regions, a shift from PEP to application of routine pre-exposure vaccination, especially for vulnerable children and old people, should be considered. This approach is expected to effectively and dramatically reduce the incidence of rabies cases, although a prompt and simplified PEP is still needed after pre-exposure rabies vaccination for complete protection after rabies exposure. However, rabies remains endemic to many parts of the developing world where the resources of appropriate PEP are limited, the infrastructure and facility are inadequate, and, most importantly, awareness about rabies is lacking. In these cases, inexpensive, safe, and effective vaccines are urgently needed. This situation is even more pronounced given the fact that the most important and probably the only practical way to control rabies globally is the mass vaccination of dogs as well as wildlife reservoirs. To accomplish this goal, efficacious oral vaccines that can be given in reduced or single doses should be developed to be cost-effective.

In contrast to the steady improvement in the availability and quality of cell culture-derived rabies vaccines and RIGs, there has been a consistent lack of interest in developing antiviral therapy for patients who have missed the deadline for valid vaccination or whose symptoms have already manifested. As of now, no effective antiviral therapy is available for these patients and death is inevitable, in most instances accompanied by agonizing pain. Fortunately, recent advances in rabies treatment have generated promising tools for preventing and eradicating rabies, at least in murine and non-human primate models. These developments provide hope for realizing the goal of curing patients who are suffering from rabies.

The rationale for rabies therapy is that RABV may stay at the entry site for days or weeks before reaching the CNS and causing symptoms, providing valuable time to limit and control the virus infection. Of the developments in rabies therapy, live-attenuated virus based vaccines might be the most promising. For a successful PEP, vaccines should rapidly induce potent protective immune responses and ideally would at the same time require less or no RIG. Live-attenuated virus-based vaccines match all these requirements and hold the promise to be curative. Most of the concerns about the use of live-attenuated virus-based vaccines come from the assumption regarding the potential risk of residual virulence and toxicity, instability, reversion to pathogenic wild-types, or recombination in nature. However, with the development of modern technology, these live-attenuated viruses used for vaccines are generally replication-deficient or restricted; their pathogenicity is commonly maximally or completely abolished and, after systemic manipulation of their genome, these viruses are theoretically highly stable and irreversible and virulence restoration could be effectively avoided. Currently, a number of live-attenuated virus based vaccines for human use have been widely used, and current data have firmly demonstrated that rabies vaccines based on live-attenuated RABV or HAdV-5 caused no adverse events or safety concerns [141,142]. Nevertheless, as with any genetically modified agent, enhanced surveillance and ecological monitoring are necessary to evaluate the relative risks of the environmental release of such viruses, given the possibility of recombination and genetic material exchange in nature, even if this is a very rare event.

Development of other novel non-viral biological treatments for rabies has also been progressing rapidly. Within the near future, there should be an increase in the number of available rabies biologics, especially with our deeper understanding of the pathogenesis of rabies and the interactions between viral and host proteins. These advancements have led to novel modalities based on gene transfer technology such as protein subunit and peptide vaccine, nucleic acid-based vaccines, and small molecules that function to interfere with the replication/spread of the virus. Nevertheless, as these forms of vaccines might result in delayed immune responses because of antigen expression/accumulation and immunogenicity, for efficient PEP, proper adjuvants would probably be needed to increase and accelerate the immune responses elicited by these vaccines. However, given the ability to elicit potent and broad immune responses, the excellent safety profile, and the rapid response to newly emerging pathogens of nucleic acid-based vaccines, especially RNA vaccines, the prospects for their application as therapeutic rabies vaccines are very promising. Moreover, research on siRNAs targeting RABV essential genes has also gained a lot of potential in terms of delaying the process of RABV infection and allowing PEP to take effect. Recent progress on proper delivery systems has potentiated the future use of not only siRNA but also other therapeutic molecules such as small-molecule drugs [143] to cross the BBB for rabies treatment. Furthermore, a lot of interest has been focused on the development of monoclonal antibodies as potential alternatives for the limited and expansive RIG and, given the rapid advances in antibody engineering, it can be envisioned that in the near future some monoclonal antibodies would be licensed as adjuncts for RIG. As a novel form of monoclonal antibodies, BsAb has demonstrated powerful application in cancer immunotherapy and warrant further investigation in rabies treatment given their potentially noninvasive BBB penetration. Ongoing preclinical and clinical studies on these novel approaches would certainly shed light on future rabies treatment.

Antiviral therapy is an important component of future efforts to develop effective therapy for human rabies. We have never been so close to controlling and even curing rabies. Within the next few years, the burden of human and canine rabies will decrease in many developing countries and mortality due to rabies will be effectively brought down. However, it should be noted that there are still many open questions in rabies therapy. If future anti-rabies therapies are developed, a relevant animal model is needed for evaluation. Moreover, the genetic and antigenic diversity of different RABV strains with sequence differences might negatively compromise these therapies. Current rabies interventions are mainly focused on viruses from phylogroup I and future vaccine development should take other members of Lyssavirus into consideration to create a vaccine with broad-spectrum protection efficacy. These pan-lyssavirus vaccines could be realized through recombinant viruses coding for the G protein of several lyssaviruses, RABV coding for additional Gs from different lyssaviruses, or chimeric G combined from different lyssaviruses, and are thus expected to provide protection against other members of the lyssavirus genus. Moreover, as RABV infection causes severe neuronal dysfunction and injury, it is equally important to ameliorate the neuronal damage induced by viral infection as well as clear RABV from the CNS for complete recovery from rabies.

Lastly, it is important to note that while some promising results have been achieved in animal models that PEP (with live-attenuated virus-based rabies vaccines) were effective, even when administered late after challenge infection, these treatments were largely prophylactic. Therefore, these treatments have not been shown to be curative, as the animals were not exhibiting any clinical signs at the time when the treatment was performed. Currently there is no solid, scientific evidence for any curative interventions. More studies are needed to further investigate the potential of curative interventions for clinical use. In the future, appropriate combinations of a rabies vaccine, antiviral drugs, immunotherapy, and neuroprotective therapy could profoundly reshape rabies treatment and benefit more patients suffering from rabies.

## Figures and Tables

**Table 1:** A list of viruses that have been engineered to express rabies virus (RABV) glycoprotein (G) protein to act as vaccine candidates for protecting against lethal rabies.

Virus	Genus	Reference
RABV	*Lyssavirus*	[9,28]
VSV	*Vesiculovirus*	N/A
PIV5	*Rubulavirus*	[29,30]
NDV	*Avulavirus*	[31]
ORFV	*Parapoxvirus*	[32]
Vaccinia virus	*Orthopoxvirus*	[33,34,35]
HAdV	*Mastadenovirus*	[36,37]
PRV	*Varicellovirus*	[38]
AcMNPV	*Alphabaculovirus*	[39]

N/A: not available.

**Table 2:** Summary of experimental live RABV-based vaccines with potential to protect against lethal rabies.

Strain	Strategy	Target
HEP-Flury/SPBN	Deletion of the P gene	P
SAD	Insertion of an internal ribosome entry site to control P gene transcription	P
RC-HL/SPBN	Deletion of the M gene	M
SPBN	Mutation of the PPEY motif	M
SPBN/Flury-LEP/HEP-Flury	Overexpression of the G gene in the G and L gene interval	G
SPBN	Deletion of the cytoplasmic domain of G protein	G
ERA/SPBN/SAG	Point mutation of the G gene	G *^a^*
HEP-Flury	Insertion of an IFN-α gene in the G and L gene interval	IFN-α
SPBN	Insertion of an IFN-β gene in the G and L gene interval	IFN-β
SPBN	Insertion of an IFN-γ gene in the G and L gene interval	IFN-γ
SPBN	Insertion of a TNF-α gene in the G and L gene interval	TNF-α
SPBN	Insertion of a cytochrome *c* gene in the G and L gene interval	cytochrome *c*
HEP-Flury	Insertion of a MIP-1α gene in the G and L gene interval	MIP-1α
LBNSE	Insertion of a GM-CSF gene in the G and L gene interval	GM-CSF
LBNSE	Insertion of a Flagellin gene in the G and L gene interval	Flagellin
ERA	Insertion of a GnRH gene in the G and L gene interval	GnRH

TNF-α, tumor necrosis factor α; MIP-1α, macrophage inflammatory protein 1α; GnRH, gonadotropin-releasing hormone; *^a^* The point mutation of G gene strategy is always used in the form of expressing multiple copies of the mutated G gene or coupled with gene rearrangement strategy.
